# Considering the ethics of live tissue training in trauma surgery

**DOI:** 10.1136/jme-2023-109761

**Published:** 2025-03-05

**Authors:** Cara Swain, Rory Rickard, Klas Karlgren, Gert Helgesson

**Affiliations:** 1 Department of Learning, Informatics, Management & Ethics, Karolinska Institutet, Stockholm, Sweden; 2 Academic Department of Military Surgery & Trauma, Royal Centre for Defence Medicine, Birmingham, UK; 3 Western Norway University of Applied Sciences Faculty of Health and Social Sciences, Bergen, Norway

**Keywords:** Education, Animal Experimentation, Ethics

## Abstract

‘Live tissue training’, using an anaesthetised live animal substituted for a human patient for the practice of surgical skills, is a controversial topic. Although simulator technologies have developed significantly for inclusion in many areas of surgical education, it is contested that training to manage traumatic injuries requires a model that can bleed and has a dynamic circulation. This article uses the published literature to explore the values at stake regarding live tissue training in the context of trauma with the aim of considering whether such training is ethically justifiable, to any degree. We present criteria for the ethical evaluation of live animal use in trauma simulation alongside descriptions of the pro- and contra-arguments present in the literature. Our conclusion is that justification is challenging and must be considered on a case-by-case basis—it is important that the difference gained from using a live animal compared with the best alternative simulator has to be greater than the clear ethical downside of using animals.

## Introduction

 Training in the craft specialty of surgery, with a focus on practical skills, combined with applied knowledge, decision-making processes, communication and teamwork, requires regular and sustained practice. While theoretical knowledge in surgery is usually obtained through visual and textual material and didactic lectures, hands-on learning is mainly obtained through clinical practice, augmented by various forms of simulation-based training. Examples of simulated training include use of computer-based training, virtual reality, anatomically correct models of organs and other physical training products for specific skill tasks, full body mannequins, animal and human cadaveric material, and live animals.^1–3^ The significant growth of simulation in surgical education is reflective of the transition in surgical training away from the traditional apprenticeship model. This shift is due to a combination of patient safety concerns, rapid advances in surgical techniques and technologies, reduced working hours for trainee surgeons, increased student numbers, decreased patient availability, and increased patient and public expectations.^4–6^ Therefore, it has even been argued that proper and careful development of simulation-based medical education is an ethical imperative.^7^

So-called ‘live tissue training’ (LTT) involves the use of an anaesthetised live animal as a simulated patient, typically for the educational practice of acute and emergency care and procedural training. Within the field of surgery, animals have been reported to be used in various specialties (neurosurgery, cardiothoracic, abdominal, trauma and reconstructive surgery, etc.) to practise diverse techniques including endoscopy, laparoscopy, microsurgery, and robotic and open surgery.^5^ When learning to manage emergency trauma, the anaesthetised animals are inflicted with traumatic injuries, such as a gunshot or a stab wound, in order for medical students, trained physicians or other healthcare professionals to learn to manage the consequences of those injuries.^3^ Historically, LTT has been used in the context of military trauma education^8^ and has been seen as an explanatory factor in the improved success rates of managing injuries caused in combat.^9 10^

In recent years, however, the practice of using live animals for medical training has become increasingly questioned^3 5^ and contended in the scientific literature, particularly within the ‘letters to the editor’ sections of journals.^11–20^ LTT has been considered an ethically unacceptable practice due to its infringement on animal rights and effects on animal welfare. It has, furthermore, been argued that the very use of the expression ‘live tissue training’ contributes to sanitising and, in effect, hiding the truth—that sentient animals are being used as human patient substitutes.^12^ Live animal use has decreased,^21^ particularly in training to manage trauma in the emergency setting.^22–24^ In the light of technological advances in medical simulation, it has been questioned whether live animals are even needed from a learning perspective, considering available alternative training methods.^2 7^

This article will explore the values at stake regarding LTT in the context of trauma with the aim of considering whether such training is ethically justifiable, to any degree. No original empirical research is presented; this work does not involve any sensitive personal data, as understood by the European Union’s General Data Protection Regulation (GDPR), and it does not require ethical review according to the Swedish Ethical Review Act (SFS 2003:460). This article will first present the theory-informed ethical criteria for evaluation, followed by the arguments for and against LTT as reported within the published literature. Finally, an overall judgement based on the evidence will be provided as to whether LTT can be justified to some extent or if it should be altogether abandoned.

## Ethical evaluation

### Input from ethical theory

There are different possible positions on the ethical relevance of the use of animals in surgical education. Some would say that LTT is wrong, hence unacceptable, regardless of its pedagogical value to improve surgical skills. Others might argue that animal considerations are important but not decisive on their own. Here, we consider the utilitarian and deontological positions in relation to this topic.

#### Utilitarianism on use of animals

Consequentialist ethical theories value actions in terms of their consequences. Utilitarianism is the most well-known consequentialist theory, which in its most commonly defended form, is an ethical theory saying that well-being is of singular importance. The greater the net total amount of well-being (in terms of increasing happiness and reducing suffering) attributed to an outcome, the better that outcome is. According to utilitarianism, animals matter ethically only in the sense that their welfare (well-being) must be considered. When making comparisons of alternative options, both direct and indirect effects on those affected by the decision are to be considered. The action that should be taken is the one realising an outcome containing at least as good a balance of positive well-being over pain, suffering, anxiety, disappointment, etc as any other option.^25^ Utilitarian calculations add up well-being effects on those affected in each alternative action to a total sum. Alternative actions are then compared in terms of total sums, where the alternative with the greatest overall value is the best action.

In utilitarian thinking, the use of animals in LTT is justified if the overall effect of the use is greater in terms of well-being compared with refraining from its use (hence avoiding both its positive and negative effects) and if there is no other training option the use of which would be superior to LTT (with its pros and cons). However, what matters to utilitarianism is not the educational value of trauma training on live animals per se but the consequences such training has in terms of well-being—by reduction of pain, suffering, and loss, happiness of seeing injured relatives receiving good care, and satisfaction of a good surgical outcome, but also by the increased capacity for well-being-productive actions by those being the true beneficiaries of the surgical training, that is, surgical patients treated by surgeons and medics having undergone the training.^i^

If the effects of educational programmes on learning are difficult to measure, the overall effect of training programmes for all sorts of outcomes in society, or on the battlefield, are even more so. For instance, according to utilitarian theory, the value of LTT depends, among other things, on the degree to which it is put to practice. That is, if a group of surgeons who received LTT then put those skills to use on 100 patients, the added value of the training to outcome is a mere tenth of the value of the skill equally well put to use on 1000 patients. This does not matter when simply comparing the quality of training models, but it is relevant for the justification of use of animals in trauma training since the magnitude of the benefits is obviously relevant to the overall outcome in terms of benefits and harms. A general implication of this kind of reasoning is that sufficiently large numbers benefited can, in principle, always justify the use of sentient beings as merely means, exposing them to pain, stress or premature death. For instance, if the disvalue of using an animal in LTT is 50 times greater than the average benefit to the patient operated on by someone who had undergone this training, then the use of the animal is nevertheless justified if more than 50 patients are treated. If the disvalue is 100, treatment of more than 100 patients is required to justify LTT and so forth.^ii^

#### Deontology on the use of animals

Not everyone will accept that the loss of animal lives can be counterbalanced by future surgical benefits. Alternative positions^26–28^ to this total-value-of-consequences view are those defending the idea of ethical principles providing restrictions on actions, for instance because human beings and animals have moral rights that need to be respected. The idea of ethical restrictions on actions means that some options are in themselves bad and should be avoided.^29^ According to ethical theories condemning murder, unprovoked violence, kidnapping, threatening behaviour, etc in this manner, such actions are impermissible in themselves, not because of their negative consequences. If these principles are given the strictest interpretation, they state that such alternatives must always be avoided, even if that would lead to a less-than-optimal overall outcome in terms of well-being, since they trump any option allowing the wrongs to take place. Even if (some of) the principles proposed were such that they do not necessarily trump any other consideration, they would still provide other reasons to consider than consequences in terms of well-being.

Imagine it was suggested that LTT should be carried out in the near future using human beings as training objects. Most citizens would probably find this suggestion outrageous and unacceptable and would probably not be appeased by guarantees from those proposing it that no human participating as a training object would feel anything when being inflicted with physical harm, surgically operated on and later euthanised. Those defending the idea of animal rights take a similar stance regarding this kind of use of animals. Some argue that animals should not be regarded as property or treated as resources for human purposes at all.^28^ From this kind of view, avoidance of pain and suffering is not enough, the idea being that there are certain things you simply must not do to sentient beings, or at least things you mustn’t do on the mere basis that they are justified based on a utilitarian calculation. If it were the case that LTT was essential to future successful trauma treatment, and LTT involved absolutely unacceptable use of animals, then LTT must be avoided at the price of future failure of trauma treatment, according to this way of reasoning.

#### Conflicting outlooks

A take-home message from this quick, and admittedly superficial, visit to ethical theory is that it is not obvious that the sole consideration we need to take regarding the handling of animals is their well-being. The different theoretical outlooks provide drastically different responses. If animals must be respected as individuals similarly to what is expected vis-à-vis human individuals, then LTT must be stopped regardless of its educational value, simply because it is unacceptable to expose animals to such treatment for such purposes. On the other hand, if we follow utilitarianism and regard well-being as the overall measuring rod, then LTT is acceptable if defensible in terms of total well-being effects, as long as animals are treated well and are properly sedated before being physically harmed, operated on and killed.^iii^

### Criteria for evaluation

The discussion throughout this article of the relative benefits of different simulation models for trauma training is primarily relevant to a consequentialist outlook. Although an interesting and worthy alternative, deontological approaches comprising absolute restrictions on what is permitted with regard to the handling of animals would render any detailed discussion of the pros and cons of LTT extraneous.^iv^ In other words, if using live animals for surgical trauma training is considered unacceptable in all circumstances, then the discussion stops there—the magnitude of expected benefits and degrees of suffering are then irrelevant.

**Figure 1:**
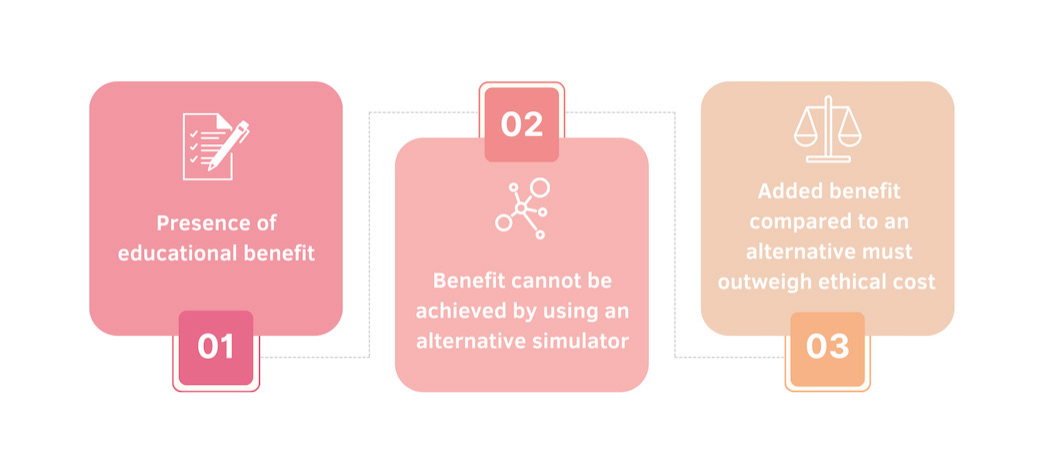
Summary of criteria required for LTT justification. LTT, live tissue training.

Our evaluation builds on a paper published by Rubeis and Steger^3^ which ethically evaluated the use of LTT in the context of trauma training. Informed by the utilitarian views of Peter Singer,^30^ they suggested two criteria by which to evaluate its use. First, LTT must have evident benefits, considering the harm and suffering caused to sentient beings. Second, there must be no equally good or better replacement for LTT among available alternative simulation models.^3^ It is worth noticing that these criteria are insufficient as stated to ethically evaluate LTT since it could be the case that there are indeed benefits from using LTT (first criterion fulfilled) and that there are no alternative models that can deliver at least as well on every point delivered by LTT (second criterion fulfilled), but that LTT is nevertheless not justified. This would be the case if the benefits (ethical gains) of irreplaceable LTT are lesser than the harms (ethical costs) caused by it. To be explicit about this, we can state the criteria that have to be met for LTT to be justified (figure 1) as follows:

1. There must be educational benefits from using LTT.
2. The benefits of LTT cannot be achieved as well or better by replacing LTT with use of other simulation models.
3. The added value of the educational benefits from using LTT, compared with other available simulation models, must outweigh the (added) ethical cost of using LTT.

Two remarks should be made at this point. First, even if replacements are superior to LTT overall, there may still be some educational benefits from using LTT that cannot be matched by other simulation modalities. This means that it may be the case that the second criterion is not generally fulfilled, but fulfilled for one or more specific educational uses, which might then justify these specific uses.^v^ Second, to iterate the remark made above: even if some aspects of LTT are irreplaceable and beneficial, it might be the case that these specific benefits are not great enough to justify the use of animals in LTT. This means that it is not enough to identify aspects of trauma training where LTT prevails to justify this type of training. The difference between what can be achieved by LTT compared with potential alternatives must be great enough to justify the use of live animals in trauma training or other surgical training. Leaving aside the profoundly problematic issue of what is the adequate unit of measurement, and just assuming we are measuring overall value and disvalue, consider the following example as an illustration of this ‘sufficient difference’ idea: if pedagogical benefits gained by using LTT are 10, benefits gained by using the closest alternative 7, and ethical costs of using animals are 5 (leaving us with the disvalue −5), then using LTT is not justified since 10–7=3, which is less than 5. Put differently, the added value of using LTT compared with the closest alternative is less than the added ethical cost of doing so.

## Material for the analysis

The literature used for the present analysis was identified in a systematic review with a pedagogical focus, to explore how educational interventions using a live animal model to train in the emergency management of trauma are designed, performed and evaluated, to better understand and characterise any educational merits of LTT. The strategic search aimed to identify empirical support from observational and interventional studies relevant to an evaluation of the continued use of LTT. During the systematic review process, papers recognised as of particular relevance to an ethical discussion of LTT were collated as they were encountered. Results from the systematic review have been published in a separate article by Swain *et al*.^31^ The analysis of the literature carried out here makes use both of the already analysed empirical papers focusing on educational merits and on papers, commentaries, etc. identified as explicitly addressing ethical issues.

## Pros and cons of LTT

Claims in the literature in support or critical of using LTT for developing or improving surgical skills are mainly found in empirical work exploring the effectiveness of using live animal models, analysed by themselves or compared with alternative modalities simulating surgery on human patients, evaluating LTT programmes, or exploring learner perceptions of LTT.^31^ These claims are mainly of an empirical nature, for instance, stating that there are (or are not) educational benefits of using a live animal compared with an alternative simulator model. In other words, such articles may provide both pro- and contra-arguments regarding the pedagogical benefits of using LTT for surgical hands-on training.

For an overview of identified pro-arguments and con-arguments, see tables 1 and 2; the subsequent sections critically discuss these arguments.

**Table 1:** Arguments in favour of live tissue training identified in the literature

	Identified arguments	Sources	Counterarguments
**1**	Learners’ preference compared with other simulation options.	Barnes *et al*, 2016^32^; Bergmeister *et al*, 2020^5^; Bukowski *et al*, 2018^33^; Hall *et al*, 2014^34^; Prudhomme *et al*, 2021^37^; Savage *et al,* 2015^35^; Sohn *et al*, 2007.^10^	That LTT is liked by learners does not by itself support the view that it is better.
**2**	LTT is a better option in terms of patient safety in comparison with supervised surgical training on human patients.	Prudhomme *et al*, 2021.^37^ Bergmeister *et al*, 2020.^5^	Any other simulation model not using human subjects to train on is equally safe for patients.
**3**	LTT provides means to acquire and practice psychomotor skills, for example, regarding low-exposure military traumas (such as gunshot or splinter/shrapnel wounds)	Mahoney *et al*, 2020^36^; Martinic, 2011.^39^	LTT is not the only means to acquire and practice these skills.
**4**	LTT provides opportunities to practice non-surgical skills in the operating room, such as situational awareness, communication and working in/managing a surgical team in emergency situations.	Prudhomme *et al*, 2021.^37^	
**5**	Several different surgical procedures can be performed on the same animal.	Bergmeister *et al*, 2020^5^; Prudhomme *et al*, 2021.^37^	Other simulators allow for same procedure to be repeated multiple times, which may be beneficial, but not possible with a live animal.
**6**	Greatest overall realism among simulation models, for example, tissue tactility, perfusion and anatomical representation.	Ali *et al*, 2012^22^; Bergmeister *et al*, 2020^5^; Bukowski *et al*, 2018^33^; Mahoney *et al*, 2020^36^; Martinic, 2011^39^	
**7**	Realism of LTT can be enhanced if combined with realistic surroundings, for example, warlike operating theatre settings.	Jacobs *et al*, 2003^44^; Mahoney *et al*, 2020^36^; Martinic, 2011^39^; Swain *et al*, 2023.^31^	This does not apply exclusively to LTT.
**8**	Bleeding significantly contributes to realism and invokes urgency to act.	Ali *et al*, 2012^22^; Bukowski *et al*, 2018^33^; Gaarder *et al*, 2004^40^; Mahoney *et al*, 2020^36^; Swain *et al*, 2023*.*^31^	
**9**	Physiological responsiveness contributes realism due to the animal body’s responses to actions taken.	Ali *et al*, 2012^22^; Bergmeister *et al*, 2020^5^; Bredmose *et al*, 2021^43^; Bukowski *et al*, 2018^14^; Da Luz *et al*, 2015^14^; Izawa *et al*, 2012^42^; Mahoney *et al*, 2020.^36^	
**10**	LTT is experienced by many to have a larger emotional impact than other simulation models, contributing to learner engagement.	Bukowski *et al*, 2018^33^; Daly *et al*, 2014^21^; Mahoney *et al*, 2020^36^; Swain *et al*, 2023.^31^	
**11**	Succeeding in these circumstances builds confidence and sense of self-efficacy.	Bukowski *et al*, 2018^33^; Galloway, 2000; Mahoney *et al*, 2020^36^; Swain *et al*, 2023.^31^	It is not clear whether LTT achieves this to a greater extent than alternative simulator models.

LTT, live tissue training.

**Table 2:** Arguments against live tissue training identified in the literature

	Identified arguments	Sources	Counterarguments
1	Animal ethics—use of animals in this manner is wrong.	Ali *et al*, 2012^22^; Bergmeister *et al*, 2020^5^; Bilello *et al*, 2021^23^; Bukowski *et al*, 2018^33^; Daly *et al*, 2014^21^; Martinic, 2011^39^; Prudhomme *et al*, 2021^37^	Some deontologists would disagree with this argument.
2	Lack of evidence that LTT is better than alternatives (since LTT involves injuring and killing animals, it should be avoided).	Da Luz *et al*, 2015^41^; Goolsby *et al*, 2017^46^; Hart *et al*, 2016^51^; Swain *et al*, 2023.^31^	
3	Alternative simulation models are superior or at least equivalent.	Bilello *et al*, 2021^23^; Lairmore & Ilkiw, 2015^1^; Tobin *et al*, 2017.^49^	
4	Lack of realism due to differences in anatomy, tissue thickness, texture, requirement for different drug dosages, etc make LTT less appropriate for surgical training preparing for trauma treatment of human beings.	Ali *et al*, 2012^22^; Da Luz *et al*, 2015^14^; Bukowski *et al*, 2018^33^; Bredmose *et al*, 2021^43^; Combes, 2013.^45^	Although these aspects reduce realism, LTT still provides most realism.
5	Confounding physiological effects of anaesthesia	Bukowski *et al*, 2018^33^; Combes, 2013^45^	This is a minor disadvantage; the mammalian response to trauma remains the same.
6	Inability to repeat procedures as an animal body can only be used once and can die prematurely.	Ali *et al*, 2012^22^; Bergmeister *et al*, 2020^5^; Bukowski *et al*, 2018.^33^	
7	Higher economic costs in terms of money, time, faculty, and logistics required to run an animal facility in comparison to alternative simulators.	Ali *et al*, 2012^22^; Bergmeister *et al*, 2020^5^; Bilello *et al*, 2021^23^; Daly *et al*, 2014^21^; Prudhomme *et al*, 2021.^37^	
8	Decreased humanity towards animals	Combes, 2013^45^	It is not clear whether this is unavoidable.

LTT, live tissue training.

### Arguments in favour of LTT

#### Preferred modality

The literature demonstrates that the majority of learners in several studies prefer LTT over existing alternative simulators.^10 32–35^ This may be explained by their belief that training using live animals is the best option, and indirectly indicate training quality. However, that LTT is the preferred choice or is judged to be better than available alternatives does not provide any direct support for the claim that LTT is, in fact, better. Learners who prefer LTT may be wrong about its quality—perhaps they would have gained an increased amount of knowledge, acquired a skill to a more complex stage or learnt more efficiently from other simulation models, even if they do not believe that to be the case. Also, since people generally want to do worthwhile and ethically justifiable things, their responses may be due to rationalisation after the fact.

#### Unique learning opportunity

There is a colloquial argument that LTT provides a unique learning opportunity^36^ and several arguments in favour of LTT contain claims of uniqueness. The claim that LTT is unique, in the sense of providing ways to learn things in relation to trauma surgery that cannot be learnt in other ways, is contested by those arguing against continued use of LTT. Some have suggested that a clear advantage of LTT is that it provides the opportunity to practice several different surgical procedures on the same animal.^5 37^ This is an advantage compared with artificial simulation models designed for the practice of one specific task, but there are products on the market that similarly allow the training of several different procedures on the same mannequin,^38^ so in comparison with these LTT offers nothing unique in this regard.

Another argument states that LTT is a means for the individual to acquire and practice psychomotor skills, for instance, to compensate for limited prior exposure to traumatically injured patients. Since other simulation models, for example, human cadavers or cut-suit task trainers, also provide the opportunity to acquire and practice psychomotor skills, this does not provide an argument for general use of LTT to acquire and improve such skills. However, to the extent that LTT focuses on specific and rare traumatic injury patterns, for instance, ballistic and blast injuries sustained in combat, it could be the case that LTT offers something unique, or at least something unusual enough not to be covered by alternative simulation models. In principle, this lack of realistic alternatives could be remediated by the invention of novel artificial simulation models, but in their absence, LTT might be a feasible option for certain learner populations, if these specific skills to be trained are of sufficient importance to justify the means to achieve the relevant skills. At least this argument cannot be dismissed without further considerations of what is gained and at what cost.

#### Realism

An argument stressed in several reports concerns superior realism of LTT compared with other simulation modalities, for instance, regarding tissue tactility, perfusion and anatomical representation.^5 37 39^ More specifically, bleeding has been argued to add strongly to realism.^36 40^ People who experience serious traumatic injuries bleed, and bleeding makes a scenario more difficult to manage from both a sensory (visual, tactile) perspective and regarding stress for the patient and clinician(s). Furthermore, bleeding contributes to time pressure, a ticking clock in the sense that blood is a finite resource and exsanguination will lead to organ failure and death if not managed urgently and appropriately. As commented by Gaarder *et al*: ‘What makes the live porcine model superior to other simulated models is the live tissue feeling combined with the presence of real haemorrhage that does not stop unless controlled’.^40^

Physiological responsiveness has similarly been identified as an aspect adding to the realism.^36 41–43^ In the presence of bleeding, the animal’s heart rate will increase and the blood pressure will decrease; as the source of the bleeding is controlled, these parameters should improve. Bredmose *et al* commented that appropriate and accurate sensory feedback alongside physiological feedback from the animal simulator in response to actions taken by the learner is important information, reflective of a similar situation in real life. While dissimilarities are problematic to some extent, training on artificial models or computer-based training cannot provide anything resembling in vivo training as regards ‘tissue feeling and tactile sensation’.^43^

The experience of realism can be enhanced further by situating the animal in realistic surroundings, like a battlefield operating theatre or in the environment of a military field exercise. Contextualising where the surgery takes place appears and feels realistic (influenced by relevant sounds, smells, stressors), mimics relevant real-life situations and potentially makes the real-life experiences seem more familiar when first encountered.^36 44^ The context of an operating theatre, including relevant sounds and smells, may, of course, be introduced to training situations involving alternative simulators such as mannequins or live actors wearing ‘cut-suits’ to increase overall realism, so this opportunity cannot be considered unique to LTT.

#### Emotional impact

Importantly, the argument goes, the realism of the LTT has a clearly larger emotional impact than alternative simulation models, such as mannequins. The fact that the training deals with a life, and hence with risk of death due to failed procedures, in combination with perceptions of smells and touch and the urgency underlined by bleeding, influences those involved in LTT emotionally. Strong sudden emotions may affect those practising in unexpected ways, such as ‘freezing’ and being unable to act as a consequence of being overwhelmed by the situation.^10^ Hence, there are emotionally impactful experiences to learn from that will not occur in other modes of simulation.^31^

An additional realism argument claims that LTT provides opportunities to practise non-surgical skills that are also of great importance in the operating room, relating to communication and working in a surgical team in an emergency situation or having the responsibility to manage it.^37^ Again, it is possible to train these particular skills without using a live animal as a simulation modality. Mannequins have been used for decades for such training, so there is arguably no specific advantage to using LTT for these purposes.

### Arguments against LTT

#### Animal rights and well-being

The main argument against LTT is normative and relates to the rights and well-being of animals, as discussed previously. As a clarification to criticism of LTT relating to animal suffering, it should be noted that the use of animals for LTT follows strict protocols and is typically overseen by certified veterinarians and personnel trained for the purpose.^45^ The animals should not experience any pain in relation to the training since they are deeply anaesthetised in advance, given analgesia prior to wounds being inflicted and remain monitored under anaesthesia throughout the training session. On completion of training, or if they exhibit signs of distress, the animals are euthanised.^39 45^ Pain may, therefore, not be the main ethical issue here, although it is easy to believe it would be when disregarding existing requirements on acceptable use.

#### Lack of evidence in support of LTT

Apart from the animal rights and well-being arguments, perhaps the most important argument against LTT is the lack of, or limited, evidence in its support. When referring to previous systematic reviews, Swain *et al* noted the overall conclusion that ‘the body of evidence is poor to moderate, and neither adequate nor robust enough to determine whether LTT is superior to other simulation methods’.^31^ Besides, studies designed to investigate the matter are heterogeneous, which makes systematic comparisons difficult,^41 46 47^ and there is an absence of support for greater long-term effects of this type of training. Alternative simulator models are available,^38 48^ and some studies suggest that these alternatives provide equivalent or superior learning outcomes compared with LTT^1 42 49^ for certain prehospital trauma skills.^34 35 50 51^ Where research has been conducted, differing interpretations of results by individuals have led to polemic commentary in the literature, for instance, Dua versus Hall,^15 20^ Crandall versus Barnes *et al*,^11 13^ Green versus da Luz *et al*.^14 17^

A reason why this is weighty argumentation against LTT is that a case can be made that the burden of proof lies with those conducting or favouring LTT.^3^ This is the case if animals need to be considered for their own sake beyond a universal well-being calculus, and perhaps also if such a calculus is the only moral measuring rod. There is an existing disadvantage and moral cost associated with LTT, namely the use of animals till their death. If this expense leaves us at a negative position on a scale ranging from bad to good, then a lack of awareness of the benefits of LTT implies that it is impossible to judge potential positive movement along the scale away from that negative position. To continue with LTT, there should be some clear indication of positive effects not attainable by use of other simulation models. As noted by Rubeis and Steger,^3^ there is a general tendency in the literature to think that the burden of proof lies with those who suggest that LTT should be abandoned. The argument is that surgeons and medics are doing well, so why make a change in the absence of proof that nothing is lost in the process? But this argument assumes what needs to be proved, namely that LTT explains the success.

#### Anatomic limitations and costs

Another argument against LTT concerns anatomic limitations of animals. Pigs are used because of their approximate size and similar anatomy to the human torso. Alternative anatomy and finer details, such as the toughness of tissues and size of arteries, reduce the realism experienced by the learner when operating on an animal and may negatively influence training potential by introducing erroneous learning relating to tactile feedback.^31^ Very few supporters of LTT question this argument—it is clear that the anatomy of an animal does not accurately represent the anatomy of a human, but this is felt to be of lesser concern than the physiological realism obtained from an alive and dynamic circulation.

Many agree that economic costs are a motivator for moving from LTT to other simulation models—training with live animals is expensive.^21 23^ Procurement of animals varies in cost, but there are significant costs associated with managing an animal institution, licensing, employment of appropriately qualified veterinary staff and consumable equipment. Whereas the initial financial outlay of non-animal simulation mannequins, for example, may be significant, associated costs are minimal. A further advantage of many non-animal simulation models is the opportunities to repeat. Non-animal simulation models can provide opportunities for training repetition at significantly lower logistical and economic cost compared with use of LTT.^5 21 23^

## Implications for use of LTT

In conclusion of this presentation of arguments for and against the use of LTT, much is contested when it comes to which type of simulation modality is better than others and how use of a live animal fares in this competition. There are potential, but not verified, advantages with LTT relating to unique practice opportunities of rare and unusual trauma situations and to the realism of the training, which covers several aspects. But as we argued above when discussing criteria for valuation, it is not enough that live animals prove to be the best simulation model in certain respects; this added value must be justified in terms of the use of animals.

From the descriptive input and arguments in the literature, it does not seem justifiable to use LTT at all levels of training (novice to expert) and for all training objectives. For instance, learning basic surgical skills, such as making an incision or suturing a wound, can be done on inert materials, ex vivo animal tissue or cadavers; there is negligible added value of using live animals. Also, many targeted aims can be achieved while avoiding LTT, such as acquiring trauma procedural skills of cricothyroidotomy and chest tube thoracostomy. One military researcher stated that they ‘should make the move away from all animal simulation when effective equivalent artificial simulators exist for a specific task. For emergency procedures, this day has arrived’.^20^ A report published by NATO (North Atlantic Treaty Organisation) concluded that ‘the use of the live tissue model for training medical personnel should not be replaced, but rather the use of this model should be reduced and the techniques refined’.^2^

For more complex clinical situations, training involving live animals may add value ranging from ‘very standardised procedures such as microsurgical vessel anastomosis to complex simulations involving transplantation, intestine surgery, intra-abdominal bleeding complications.’^5^ There are also arguments for training that includes multidisciplinary teams to optimise teamwork in critical situations, but it is hard to say whether LTT adds enough value to such training to justify it.

If LTT is to be used, an ethical requirement is that it must be thoroughly considered for each potential use whether there in fact is something important to learn from the use that cannot, in any vein near that of what is accomplished through LTT, be gained through other means. If it is possible and likely to achieve similar or greater skills through the use of other simulation models, at similar or lower moral costs, then LTT cannot be justified. This argument is equivalent to the replacement argument typically proposed in relation to the use of animals in research.^5 37^ Education should, hence, be planned such that LTT, if used, is the end-point practice before moving to participation in surgery on actual patients. Before that, practice should take place on computer models, artificial models, dead animals, or human cadavers, or several of these.^2^ These other means of training surgical skills need to make up the main volume of training opportunities in order for the LTT to be dedicated to the added value of such training, as compared with basic skills.^37^ Technological and functional improvements in other simulation models may further decrease the justifiable use of LTT.

## Conclusions

Our overall conclusion regarding use of animals for training in trauma surgery is that it remains unclear whether such use is justified or not. Difficulties to settle the issue partly depend on the fact that different theoretical outlooks provide very different answers. But unclarity remains also within an ethical outlook focusing on the value of the consequences. Some uses are potentially justified, because they provide something unique or otherwise very hard to achieve, due to greater realism. Several other uses are clearly not justified if animal interests matter. If LTT is to be justified, it is ethically required that each potential use is thoroughly considered regarding whether there in fact are gains to make that cannot be achieved by use of other simulation models. Furthermore, that LTT is superior in some respects or contexts to other simulation options is not sufficient to justify its use. The difference gained from using LTT compared with the best alternative simulator has to be greater than the ethical downside of using animals for such training. If animal suffering is adequately handled, the main objection to LTT regarding the use of animals must concern animal rights rather than animal well-being.

## Data Availability

No data are available.

## Footnotes

Admittedly, there may be other beneficiaries, for example, surgeons if their well-being is increased (either through the sense of greater competency or through effects of actually becoming more competent) and relatives, colleagues and friends of patients receiving better care.

We are not making any suggestions here regarding what values to enter into the calculation, but merely describing this way of thinking. If the disvalue of using animals would instead be 1000 or 10 000, there would still be a number of patients helped that would justify LTT.

These conditions are not added because disregarding them would be inherently unacceptable to utilitarianism, but because the overall outcome would generally be better if they were followed.

The deontological or rule-based approach to ethics very briefly sketched here actually divides into two very different views regarding respect for animals. Apart from deontologists arguing in favour of animal rights,^28^ there are deontologists arguing that animals have no moral standing; they have no rights or morally relevant interests whatsoever that needs to be considered.^27^

For this to be the case, it is not enough that some aspect of LTT cannot be met by alternative simulation models, such as perceived level of realism by learners. For LTT to be justified in any specific educational context, it must be superior overall in that specific context. However, even if there is always some alternative simulation model that is superior overall regardless of what is trained, perceived realism on a level exclusively delivered by LTT may nevertheless be indispensable for a good education in trauma surgery, which would then imply that it should be included somewhere in the education package, provided criterion 3 is fulfilled.

## References

[1] Lairmore MD, Ilkiw J (2015). Animals Used in Research and Education, 1966-2016: Evolving Attitudes, Policies, and Relationships. J Vet Med Educ.

[2] RTG-HFM-242 (2020). Technology alternatives for medical training: minimizing live tissue use.

[3] Rubeis G, Steger F (2018). Is live-tissue training ethically justified? An evidence-based ethical analysis. Altern Lab Anim.

[4] Agha RA, Fowler AJ (2015). The role and validity of surgical simulation. Int Surg.

[5] Bergmeister KD, Aman M, Kramer A (2020). Simulating Surgical Skills in Animals: Systematic Review, Costs & Acceptance Analyses. Front Vet Sci.

[6] Strong VE, Forde KA, MacFadyen BV (2014). Ethical considerations regarding the implementation of new technologies and techniques in surgery. Surg Endosc.

[7] Ziv A, Wolpe PR, Small SD (2006). Simulation-based medical education: an ethical imperative. Simul Healthc.

[8] Knudsen PJ, Darre EM (1996). Training in wound ballistics: operation exercise at the Defence Medical Training Centre. J Trauma Acute Care Surg.

[9] Butler FK, Blackbourne LH (2012). Battlefield trauma care then and now: a decade of Tactical Combat Casualty Care. J Trauma Acute Care Surg.

[10] Sohn VY, Miller JP, Koeller CA (2007). From the combat medic to the forward surgical team: the Madigan model for improving trauma readiness of brigade combat teams fighting the Global War on Terror. J Surg Res.

[11] Barnes SL, Kerby J, Armstrong J (2017). Response to: Comment on: Live tissue versus simulation training for emergency procedures: Is simulation ready to replace live tissue?. Surgery.

[12] Crandall M (2010). “Live Tissue” is not the answer. J Trauma.

[13] Crandall M (2017). Comment on: Live tissue versus simulation training for emergency procedures: Is simulation ready to replace live tissue?. Surgery.

[14] da Luz LT, Nascimento Junior B, Tien H (2015). Current use of live tissue training in trauma: a descriptive systematic review - author response. Can J Surg.

[15] Dua A (2014). Letter in response to: “Comparison of Self-Efficacy and Its Improvement After Artificial Simulator or Live Animal Model Emergency Procedure Training” by Hall AB, Riojas R, Sharon D, Mil Med 2014; 179(3): 320-3. Mil Med.

[16] Gala SG, Balsam MJ (2017). Letter in response to: Kim M, Torrie I, Poisson R, Withers N, Bjarnason S, DaLuz LT, Pannell D, Beckett A, Tien HC. The value of live tissue training for combat casualty care: A survey of Canadian combat medics with battlefield experience in Afghanistan. Mil Med. 2017 Sep; 182 (9): e1834-e1840. Mil Med.

[17] Green PP (2015). Current use of live tissue training in trauma: a descriptive systematic review. Can J Surg.

[18] Reeds MG (2010). Live Tissue: Ideal for Trauma Training?. J Trauma Acute Care Surg.

[19] Reeds MG (2010). Trauma Training Using the Live Tissue Model. J Trauma Acute Care Surg.

[20] Hall A (2014). Letter in response to: “Comparison of Self-Efficacy and Its Improvement After Artificial Simulator or Live Animal Model Emergency Procedure Training” by Hall AB, Riojas R, Sharon D, Mil Med 2014; 179(3): 320-3 Response. Mil Med.

[21] Daly SC, Wilson NA, Rinewalt DE (2014). A subjective assessment of medical student perceptions on animal models in medical education. J Surg Educ.

[22] Ali J, Sorvari A, Pandya A (2012). Teaching Emergency Surgical Skills for Trauma Resuscitation-Mechanical Simulator versus Animal Model. ISRN Emerg Med.

[23] Bilello L, Ketterer A, Yarza S (2021). Procedural training models among emergency medicine residency programs. Clin Exp Emerg Med.

[24] Henry S, Brasel KJ, Josesph K (2018). ATLS at 40: distinguished past, bright future. Bull Am Coll Surg.

[25] Tännsjö T (2019). Hedonistic utilitarianism.

[26] Regan T (2013). The case for animal rights. Ethics, humans and other animals.

[27] Cohen C (1986). The Case for the Use of Animals in Biomedical Research. N Engl J Med.

[28] Taylor A (2009). Animals and ethics: an overview of the philosophical debate.

[29] Kagan S (1998). Normative ethics.

[30] Singer P (2011). Practical ethics.

[31] Swain CS, Cohen HML, Helgesson G (2023). A Systematic Review of Live Animal Use as a Simulation Modality ('Live Tissue Training') in the Emergency Management of Trauma. J Surg Educ.

[32] Barnes SL, Bukoski A, Kerby JD (2016). Live tissue versus simulation training for emergency procedures: Is simulation ready to replace live tissue?. Surgery.

[33] Bukoski A, Uhlich R, Bowling F (2018). Perceptions of Simulator- and Live Tissue-Based Combat Casualty Care Training of Senior Special Operations Medics. Mil Med.

[34] Hall AB, Riojas R, Sharon D (2014). Comparison of self-efficacy and its improvement after artificial simulator or live animal model emergency procedure training. Mil Med.

[35] Savage EC, Tenn C, Vartanian O (2015). A comparison of live tissue training and high-fidelity patient simulator: A pilot study in battlefield trauma training. J Trauma Acute Care Surg.

[36] Mahoney A, Reade MC, Moffat M (2023). Experiences of medical practitioners in the Australian Defence Force on live tissue trauma training. *BMJ Mil Health*.

[37] Prudhomme T, Matillon X, Dengu F (2021). Residents and patients benefit from surgical simulation on a live porcine model, could we consider it as ethical?. Prog Urol.

[38] Bowyer MW, Fransman RB, Stefanidis D, Korndorffer Jr JR, Sweet R (2019). Comprehensive healthcare simulation: surgery and surgical subspecialties.

[39] Martinic G (2012). Military 'live tissue trauma training' using animals in the US-its purpose, importance and commentary on military medical research and the debate on use of animals in military training. J Mil Veterans Health.

[40] Gaarder C, Naess PA, Buanes T (2005). Advanced surgical trauma care training with a live porcine model. *Injury*.

[41] da Luz LT, Nascimento B, Tien H (2015). Current use of live tissue training in trauma: a descriptive systematic review. *Can J Surg*.

[42] Izawa Y, Hishikawa S, Muronoi T (2016). Ex-vivo and live animal models are equally effective training for the management of a penetrating cardiac injury. World J Emerg Surg.

[43] Bredmose PP, Stave H, Eriksen M (2021). Live Tissue Training on Anesthetized Pigs for Air Ambulance Crews. Air Med J.

[44] Jacobs LM, Burns KJ, Kaban JM (2003). Development and Evaluation of the Advanced Trauma Operative Management Course. J Trauma.

[45] Combes RD (2013). A critical review of anaesthetised animal models and alternatives for military research, testing and training, with a focus on blast damage, haemorrhage and resuscitation. Altern Lab Anim.

[46] Goolsby C, Branting A, Ausman J (2017). Systematic Review of Live Tissue Versus Simulation Education for Prehospital Trauma Providers. Mil Med.

[47] Hart D, McNeil MA, Hegarty C (2016). Literature Evidence on Live Animal Versus Synthetic Models for Training and Assessing Trauma Resuscitation Procedures. J Spec Oper Med.

[48] Cherry RA, Ali J (2008). Current Concepts in Simulation-Based Trauma Education. J Trauma Acute Care Surg.

[49] Tobin NM, Chan V, Evans C (2017). Is it ethical or effective? Comparison study of live porcine and human cadaver models in resident training of invasive procedures. Acad Emerg Med.

[50] Hall AB (2011). Randomized objective comparison of live tissue training versus simulators for emergency procedures. Am Surg.

[51] Hart D, Rush R, Rule G (2018). Training and Assessing Critical Airway, Breathing, and Hemorrhage Control Procedures for Trauma Care: Live Tissue Versus Synthetic Models. Acad Emerg Med.

